# A History of the Improvements Which Practical Medicine Has Derived from Auscultation; Being the Essay to Which the Medical Society of Bordeaux Awarded Its Prize in November, 1839

**Published:** 1842-07

**Authors:** G. Peyraud

**Affiliations:** Doctor of Medicine of the Faculty of Paris, Corresponding Member of the Bordeaux Medical Society


### WESTERN JOURNAL

OF

### MEDICINE AND SURGERY.

---

JULY, 1842.

---

### ART. I.—*A History of the Improvements which Practical Medicine has derived from Auscultation; being the essay to which the Medical Society of Bordeaux awarded its prize in November*, 1839. By G. PEYRAUD, Doctor of Medicine of the Faculty of Paris, Corresponding Member of the Bordeaux Medical Society. *Quæ fundata sunt in natura, crescunt et perficiuntur; quæ vero in opinione, variantur, non augentur.*—Baglivi. Translated from the French for the Western Journal of Medicine and Surgery, by CHARLES A. POPE, M. D., of St. Louis, Lecturer on Anatomy and Surgery, Physician to the St. Louis Dispensary, &c., &c.

(NOTE BY THE TRANSLATOR.—A desire to be useful, and to employ the leisure occurring at the outset of a professional career, which might otherwise be less profitably spent, has prompted the present task.

To those physicians who are already acquainted with auscultation and the advantages which have resulted from it to practical medicine, this work will, I think, give a more clear, connected, and methodical idea of the improvements derived from the discovery of Laennec than any other yet published; while to such practitioners as are yet unacquainted with the art, and who, through ignorance and prejudice, undervalue its aid, it will, I hope, set forth arguments, and afford inducements, sufficient to incite them to acquire a knowledge of this means of explanation, which will best enable them fully to appreciate its utility.

By a glance at the contents, it will be seen that our author has surveyed the whole field of auscultation, not only as to its use in the diseases of the chest, but also in its application to cases of an entirely different character. He has, I think, treated his subject with rare ability, and the work is one of just and impartial criticism. As it is addressed to those already acquainted with auscultation, I take great pleasure in recommending to the attention of such physicians as are not yet familiar with physical examination, and who are distant from our large cities, and consequently deprived of access to extensive hospitals, *Gerhard on the Chest*, and particularly the last chapter, for facilitating them in acquiring a knowledge of the physical signs.

Relatively to the theories of the sounds of the heart, I may here mention that a new one has recently been started by M. Cruveilhier of Paris. He was called to see an infant, a few hours after birth, in whom an extraordinary malformation of the chest existed. The heart was situated fairly out of the thorax, from which it had passed through a circular opening in the upper part of the sternum, being thus as completely exposed as if the sternum had been removed, and the pericardium opened. M. Cruveilhier made many interesting and important experiments in this case, with the view of determining the seat and cause of the movements and sounds of the heart. They are too long to be reported in this place; a fuller account may be found in the Medico-Chirurgical Review (January, 1842, p. 193 et seq). He infers, however, from his observations, that "the two sounds of the heart have their seat at the origin of the pulmonary artery and aorta, and their cause in the clacking of the sigmoid valves *(du redressement des valvules sigmoïdes, préalablement abaissèes)*; and that the second sound, which coincides with the diastole of the ventricles and with the contraction of the auricles, is the result of the closing together of the same valves folded back by the retrograde wave of blood."

With regard to the manner in which I have accomplished my task, I may say that no one is more sensible than myself, of its many imperfections; and those who are aware of the difficulties inherent to every translation, and the almost impossibility of giving the various shades of an author's meaning in a language other than his own, will be the first to grant their indulgence. I have endeavored always to give the sense in as plain and perspicuous a language as possible; but that some gallicisms will appear, I think more than probable).

When Baglivi wrote the lines which serve me for a motto, he established the surest principle for separating opinions founded in nature, from mere theories and vain hypotheses. Should one of these new ideas, such as are daily arising, appear in the scientific world, come from whatever quarter it may, from one of the masters of the art, or from some obscure member, let us not be misled by the clamorous admiration of complaisant friends, nor prejudiced by criticisms inspired by envy or springing from foolish precipitation. Let us wait, for time will quickly decide its worth. If this new idea be true, and founded in nature, it will gain ground despite the bitterest criticisms, and will finally become an established and incontrovertible truth. If, however, it be false and devoid of foundation, the exaggerated praises of those who have an interest in its propagation, will not sustain it. It will be changed, altered, or modified by each of its partisans, who will think, in doing so, to mask its weak side, and it will at length fall into complete oblivion, whence it will never again be drawn, save by him who would give the history of the errors of the human mind. Indeed, examples would not be wanting to establish the truth of this principle; and, to cite only one, if Gall were to rise from his tomb, on which the weight of years as yet bears lightly, would he recognise in Phrenology, as it is now taught, the science which he thought to have founded? His successors have changed all that he did; the intellectual and instinctive faculties are no longer the same which he established; the places which he assigned them in the encephalon, have been appropriated to others; and perhaps, applying to himself the principle of Baglivi, he would suspect that he had put forth an hypothesis only: *Quæ fundata sunt in natura, crescunt et perficiuntur; quæ verò in opinione, variantur, non augentur.*

Such, however, is the principle which I boldly lay down as the touch-stone to prove the excellence of auscultation. It is known how this art was discovered. In 1816, Laënnec, whilst treating a young person for a disease of the heart, conceived the idea of exploring the beatings of this organ, by means of a few sheets of paper which he formed into a scroll, and was much surprised to hear them more distinctly than he had ever done before. He immediately felt the importance of this means of investigation, and saw in it the germ of a revolution in diagnosis, which would change the face of medicine, so far as thoracic diseases were concerned. He at once set about making a series of observations at the Necker Hospital, the result of which was the immortal work that has rendered his name so familiar. With what enthusiasm would not that same Baglivi have received it, who in 1696 so sorrowfully exclaimed: "*O quantum difficile est curare morbos pulmonum! O quantò difficilius eosdem cognoscere, et de iis certum dare præsagium! Fallunt velperitissimos, ac ipsos medicinæ principes. Tyrones mei, cauti estote et prudentes, in iis curandis, nec facilem promittite curationem, ut nebulones faciunt, qui Hippocra-*

*tem non legunt.*"—(Prax. Medicæ, lib i, cap. xii, de Pleuritide.)

More than a century elapsed after this bitter and poignant reflection of the practitioner of Rome, and the state of things continued unchanged. The difficulty attending the diagnosis of diseases of the chest was still greater than that of their treatment, which, however, presented many obstacles and dangers. Laënnec's book appeared, and so complete and prompt was the revolution effected, that henceforward the proposition of Baglivi should be reversed, and it should be unanimously agreed that it is much less easy to cure diseases of the chest, than to recognize them.

Twenty-four years already separate us from that epoch, and auscultation still continues the most beautiful ornament of modern medicine; and the labors of the numerous authors who have followed Laënnec in the new path, have only confirmed the truth of his observations, the sagacity of his remarks, and the admirable exactness of the signs which he assigned to the diseases of the lungs. Auscultation has neither varied nor changed; it is precisely what Laënnec left it; for that indefatigable observer had brought it to such a degree of perfection, that there was nothing left for others but the hope of confirming what he had taught.

Will we not find in this astonishing perfection of so useful an art, the best and most unexceptionable proof, that it was really founded in nature, and not in a false hypothesis? What more natural, indeed, than to study the sounds which the respiration engenders in the organs where this function is accomplished? As these sounds cannot be modified without the function also, do not the modifications of the normal sounds, when well studied, become valuable signs in the diagnosis of the lesions which thus involve the function? This point once firmly established, all that remained was to collect a great number of facts, and especially to seek from pathological anatomy the confirmation of the relation which was supposed to exist between the modifications of the respiratory sounds, and the various degrees of the diseases that were observed. It is this which Laënnec and his successors have done, and it is in some manner an inventory of the improvements which have resulted from it to practical medicine, that I am called on to make in answer to the wishes of the Bordeaux Medical Society.

This inventory I shall make scrupulously and conscientiously. Studying successively, and in as many distinct chapters, the different diseases of the lung and of the pleura, I will commence by showing what the labors of the different authors anterior to Laënnec had done for the diagnosis of those diseases. This exposition will be long; but it has seemed to me indispensable to the proper appreciation of the immense stride which auscultation has made in pathology on such difficult points; and the importance of this progress will be better set forth and become more evident, when we shall know in detail the errors into which the best minds have fallen, deprived of this valuable means of investigation.

Coming next to the diseases of the heart, I shall have a painful admission to make; but I shall makeit, for I do not think that an eulogium *quand même*, and absolute on all points, is demanded. I will say, then, that the results of auscultation applied to diseases of the heart have thus far been almost completely useless. Abnormal sounds have been heard, but they have not been explained, because a fixed starting-point, one admitted by all, has been wanting. The interpretation which has been given, has varied according to the idea entertained of the primary cause of the natural sounds of the heart; which furnishes us with a confirmation of the second part of the maxim of Baglivi: *Quæ verò in opinione, variantur non augentur*.

A chapter will be devoted to asthma and to angina pectoris, in which I shall demonstrate that auscultation has still rendered service to pathology, by proving that if, in certain cases, the material lesions coexisting with those diseases could be recognized, they could rarely explain the morbid phenomena which constitute them; a result extremely useful, although contrary to their *localisation*, inasmuch as it proves that there is in those diseases a nervous element which gives them their essential character.

Finally, the last chapter will be devoted to a rapid survey of the various applications which have been made of auscultation to other affections than those of the chest, and especially to pregnancy.

I ought to premise, that all I shall say, will be equally applicable to mediate or immediate auscultation. The discussions concerning the superiority of one method over the other are now exhausted, and to renew them would be veritable nonsense. The results produced by them being identical, one ought not to be preferred to the other, but they should be combined together for the greater convenience of the person who practices them. Thus the naked ear will be directly applied, if it is desired, on the convex part of the back and on the anterior portion of the chest. Recourse will be had to the stethoscope for parts the conformation of which does not permit the approach of the ear, as the axilla, or with females, in whom modesty generally interdicts every other mode of auscultation. These are points now admitted by all, and quite beyond discussion; I shall not dwell longer on them.

### CHAPTER I.—OF PLEURISY AND PNEUMONIA.

I shall speak of these two diseases in the same chapter, for prior to the time of Laennec, there was no differential diagnosis, and perhaps his most legitimate claim to universal admiration arises from his having assigned to each, symptoms as different as their anatomical characters. Let us inquire what was, before his time, the state of the science on this all important branch of pathology.

Cullen thus commences his chapter on *pneumonia or fluxion of the chest*: "It is my design to comprise under this head all the inflammations which affect either the viscera contained in the thorax or the membrane lining the internal surface of that cavity; *for no sign can serve to determine exactly the different seat of the disease, &c.*" (Pract. Med., translated by Bosquillon, chap. VI, §334). This is a frank avowal, but it was at that time the honest expression of the truth, as will clearly appear from a comparison of what nosologists have said on this subject.

I know not indeed whether I ought to analyse the symptoms that Cullen attributes to *fluxion of the chest,* since we are so well apprised that under this title he includes two diseases now so distinct. These symptoms are fever, difficult respiration, pain in some part of the chest, and cough, which he says is sometimes dry, sometimes accompanied by expectoration varying in consistence and in color, but in which streaks of blood are frequently observed. These symptoms are somewhat vague; a pain in some part of the chest is not always pleuritic; but it is certain that added to fever, difficult respiration and bloody sputa, it is pretty characteristic of a phlegmasia of the organs contained in that cavity. But what part do the lung and pleura bear in this inflammation? An answer cannot be expected from the author when he adds: "There is but little reason, therefore, for designating this disease by different names taken from the part which may be supposed to be particularly affected. The term *pleurisy* answers in all cases, and it has been very improperly restricted to mean the inflammation commencing in that portion of the pleura which lines the ribs and which is particularly affected. I do not doubt that this really happens, but at the same time I suspect that it is a rare occurrence." (Op. cit. §341.)

The translator and commentator of Cullen, Bosquillon, in endeavoring to supply his deficiency, thus establishes the differential diagnosis of pleurisy and peripneumonia.

"*Characters of peripneumonia.*—The pulse is not always hard in this inflammation; it is sometimes soft; the pain in the chest is dull, the respiration is invariably difficult, and often can be performed only when the trunk is in the erect posture; the face is swollen and of a purple color; there is a cough, commonly humid, often bloody."

"*Characters of pleurisy.*—In pleurisy the pulse is hard, the side is generally affected with a pungent pain, which is increased during inspiration. The patient lies with difficulty on his side, and the cough is very painful, at first dry and afterwards moist, often bloody." (Op. cit. note by Bosquillon, vol. i, pp. 376 and 377).

The characters here assigned by Bosquillon to pleurisy and to peripneumonia are not without importance. Thus the hard pulse has been indicated as properly belonging to pleurisy, by several authors, and among others by Baglivi, who thus expresses himself: "*Si vis cognoscere pleuritidem, præcipuam curam in natura pulsus cognoscenda reponito: pulsus durities est signum fere infallibile omnium pleuritidum; et dum obscuræ sint pleuritides, vel aliis complicatæ pectoris morbis, si duritiem (id est nimiam arteriæ tensionem vibratonemque), in pulsu deprehenderis, quamvis reliqua earum signa non adsint, pro certo habeas patientem laborare pleuritide, &c.*" (Op. cit. lib. i, cap. ix, de Pleuritide).

The softness of the pulse in pneumonia has likewise been pointed out by most authors, and Dehaën particularly gives us a very ingenious explanation of it. But a distinctive character of the two diseases, far more essential than the state of the pulse, especially as it is more easily appreciable, is the pungent pain, "sometimes limited to a part which may be covered by the finger." This local and fixed pain essentially distinguishes pleurisy from pneumonia. The difficulty of lying on the affected side, is also one of the characteristic signs of pleuritic inflammation; but is it always very easy to distinguish between it and the difficulty of respiration in other than the upright posture which Bosquillon notices in pneumonia? Lastly, I ask, are these characters, however just in themselves, sufficient to guard the practitioner against the commission of error?

A little further on, Bosquillon points out *vomica* and *empyema* as ordinary results of pneumonia. A vomica may be recognized by the continuance of the cough and dyspnœa, the impossibility of lying on the affected side, and the development of hectic fever. In case of empyema, the cough, difficulty of respiration and lying in the recumbent posture are present, there is likewise hectic fever; *the patient often feels at the same time a kind of fluctuation produced by the liquid contained in the chest.* With the exception of this last symptom, all the others enumerated by Bosquillon have very little value in the diagnosis; and does this last always exist? When Bosquillon pointed it out, was it from clinical facts that he did so, or was he not unwittingly influenced by the remembrance of the Hippocratic succussion? However this may be, the sign but very rarely exists, since we shall hereafter see in speaking of pneumo-thorax, that, in order to the success of the Hippocratic succussion, there must be in the pleura both air and pus at the same time, the only circumstances under which the latter can be agitated and made to give out the noise of fluctuation mentioned by Bosquillon.

*Finally*, he adds, *the signs of hydro-thorax are superadded to these symptoms*, and the former he enumerates as follows: "The signs which characterize hydro-thorax, are dyspnœa, pallor of the countenance and œdema of the extremities; the patient experiences great difficulty in keeping the recumbent posture, he starts in his sleep and complains of palpitations. There is a perceptible fluctuation in the chest." (Cullen, op. cit., note by Bosquillon, vol. iii, p. 293.) Apart from the fluctuation of which I have just been speaking, this symptomatology is exact; but it is insufficient. It offers no means of ascertaining in which side of the chest the effusion exists, nor its extent. Moreover, there is not one of these symptoms which is not in like manner produced by a serous effusion in the pericardium; and a diagnosis founded on these data alone, without seeking new elements elsewhere, would necessarily be uncertain, imperfect, and unsatisfactory to all.

Sydenham has treated of pleurisy and pneumonia; but he regards both as the result of an inflammatory state of the blood: "When the febrile matter throws itself on the pleura or the intercostal muscles, it ordinarily happens in the commencement of the fever, the morbific matter being yet crude, and not having had time to undergo the necessary concoction and preparation, to be evacuated by the suitable outlets.

The common cause of this occurrence is the improper use of heating remedies." (Sydenham, Pract. Med., translation of Jault, p. 249.) Of course he attached but little importance to the recognition, during life, of the development and even the precise seat of anatomical alterations, which he regarded as the consequence only, and not as the producing cause of the fever. However, he clearly indicates the painful stitch in the side as one of the essential signs of pleurisy; and his translator, M. Jault, endeavoring in a note to give precision to the signs by which the existence of adhesions between the lung and corresponding pleura is recognized, thus expresses himself: "The symptoms which enable us to judge most certainly whether there be adhesion, is when the patient can lie on but one side, without pain and with a tolerably easy respiration. The adhesion is always on the side on which the patient lies easily." (Op. cit.. note by Jault, p. 248.) M. Jault supports this opinion by two reasons: 1st, that when the patient lies on the sound side, the lung tending by virtue of its weight to separate from the costal pleura, the adhesions are put on the stretch; 2d, that in the decubitus on the healthy side, the sound lung cannot supply the unsound lung in its functions. These signs are very rational, but they can only authorize the suspicion of adhesions between the lung and the corresponding ribs; they are not sufficient to render their existence certain. We know that in investigating the effects of these adhesions and the characteristic signs by which they are revealed, Laennec discovered that their constant effect was to diminish that side of the chest which had been the seat of the pleurisy, the consequences of which they were; this furnished a pretty characteristic sign. But to dwell longer on, this subject would be leaving the question which the *programme* has so precisely limited to the improvements that auscultation has made in the diagnosis and treatment of diseases of the chest. I continue my review of the nosologists who preceded Laennec.

The two diseases we are now considering are frequently discussed in the *Practical Medicine* of Stoll. But in his constant and almost exclusive eagerness to pursue the bile, and the crude and acrid matters of the primæ viæ, the practitioner of Vienna is much more frequently engaged in the search of signs to prove their complication with a bilious state, than in looking after the characters that might serve to distinguish them. Moreover, what he says of pleurisy and bilious pneumonia observed in April, 1776, proves that he regarded them as two different degrees of one and the same malady. "This observation," he says, "is important in the diagnosis: that in bilious pleurisy and pneumonia the pain is rarely increased by coughing, or during inspiration; whilst those who are attacked with true inflammatory peripneumonia, can neither cough nor respire without a violent pain in the side. Besides this, the sputa is rarely tinged with blood in bilious pleurisy, unless it be sufficiently violent for the efforts of coughing to bring away a little blood." (Stoll, Pract. Med. translated by J. Terrier, vol. i, p. 65.) Elsewhere, speaking of the importance of the redness of the face, in distinguishing false pleurisy from true inflammation of the lungs, after having examined passages from Baillon and Hippocrates on this subject, he arrives at this conclusion: "that persons are seen affected with pneumonia, whose lungs are attacked by a true and violent inflammation, although the face be very pale, &c." (Op. cit., vol. i, page 68.) From all this does it not appear very evident, that, in his view, pleurisy and pneumonia are the same thing? are not the two words used indifferently by him, at random, so to speak, without any motive to justify the employment of one or the other? Leaving very soon the description of these diseases, Stoll, in the remainder of the passage which I am analysing, busies himself in pointing out the indications for tartar emetic, a remedy which he is known to have used almost exclusively, although he was far from regarding it as contra-stimulant; and I can legitimately conclude that he has in no wise advanced the diagnosis of these two diseases.

Morgagni has devoted two letters (the 20th and 21st) of his immortal work, *De sedibus et causis morborum*, etc., to the study of pleurisy and pneumonia, under this title: *Of pain in*

*the chest, sides and back.* The illustrious founder of pathological anatomy had opened too many dead bodies to be in danger of falling into similar errors respecting the nature and seat of these two phlegmasiæ. He is seen, in the remarks which always follow each history, endeavoring to show that the part where the patient had felt the pain, is invariably the same at which the lung is found adherent to the pleura; that in cases of peripneumonia, the pain is always gravative, whilst in cases of pleurisy it is more acute and pungent, *on account of the great number of nervous fibres which terminate in the pleura.* (Op. cit., translated by Désormeaux, vol. iii. page 305). However, when the real opinion of Morgagni on the nature of these two phlegmasiæ, is sought for, it will be found that he does not think they can exist separately, except it be to a very slight extent. We find him, indeed, in his 21st letter, accumulating authorities to prove that a pleurisy cannot cause death, without the phlegmasia extends itself to the lung. "Hippocrates, in his book *De locis in homine*, has positively placed in the lung not only the seat of peripneumonia, *but also that of pleurisy. . . . . Cœlius Aurelianus* teaches, with Praxagoras, Herophylus and Euryphontes of Gnidus, that the lung *is the part that suffers in pleuritic patients.*" Further on he cites the observations which Hoffman says were made in the hospital Santo Spirito, at Rome, by *Servius, on three hundred patients affected with pleurisy,* "*among whom he constantly saw one lobe of the lung putrified and filled with matter, whilst the pleura absolutely had no appreciable lesion, or was but slightly altered in any way.*" Still further on he cites Riviere, in whose *Sepulchretum* these words are found: "*The very violent pleurisies, which ordinarily induce death, degenerate most often into peripneumonias;*" and Triller, a most worthy physician, who assures us that generally, "*in a true pleurisy there exists not merely an affection of the pleura, as has been heretofore foolishly believed, but likewise a simultaneous alteration of the very substance of the lungs, as anatomy, the only light of medicine, clearly teaches;*" and lastly, the great anatomist Haller, who says, "*that he has never thought an inflammation of the pleura alone killed any man.*" (Op. cit., vol. iii, p. 482 and following.) He had already in his 20th letter quoted Coiter, who remarks that *peripneumonia is one of those diseases accompanied by dropsy of the chest.* (Op. cit., vol. iii, p. 339).

Thus then, according to the illustrious professor of Bologna, pleurisy and peripneumonia scarcely ever exist separately. He knows their inflammatory nature, he no longer considers them as an accumulation of febrile matter upon the chest, or as a reflux of acrid and bilious matters from the stomach to the thoracic vessels. These errors had to disappear before the broad light which his ever celebrated labors shed on the seat and nature of diseases. He did not finish his work, however, but still left something to be gleaned by his successors in the vast field of pathological anatomy, which he cultivated so successfully. We shall after a while see how much more distinct the labors of the moderns, and above all of Laënnec, have made the special lesions peculiar to each of these two maladies, at the same time that auscultation will furnish us the means of recognising in the patient the separate existence of these lesions.

To continue our review of authors:

I have shown that Cullen, Sydenham, Stoll and Morgagni considered pleurisy and pneumonia as only one and the same disease. Did I not fear uselessly to prolong this work, it would be easy to add to these venerable names in the science, those of other authors not less worthy of esteem. Thus Sarcone devotes a pretty long chapter of his *History of the epidemic at Naples in* 1764, to prove that pleurisy and pneumonia are the same thing (op. cit., translated by Bellay, vol i, p. 126 and following); and we find in the Memoirs of the Academy of Sciences for 1789 an observation by Portal, *which proves that pleurisy is not essentially different from pneumonia.*

However, an opinion so erroneous did not pass without contradiction, and in vol. ix of the *Ratio Medendi*, Dehaën is seen to contend warmly with Boërhaave, in opposition to Haller and Tissot, that pleurisy and pneumonia are two distinct diseases.

Leaving these personal disputes, let us see how Dehaën in another of his works endeavors to characterise each of them. He first asks himself what is the pathognomonic sign of pleurisy? Is it the stitch in the side? No, for hysterical patients have pains in the side, which anti-phlogistics do not abate. Is it the difficulty of respiration? No, for this is also observed in asthmatic patients. Is it fever? But all acute diseases produce that. Lastly, is it the hardness of the pulse? But the pulse is hard in phrenitis. No one, then, of these isolated symptoms, essentially characterises pleurisy; but all four united, especially if the stitch in the side be increased by inspiration, authorises us to announce its existence.

Passing to peripneumonia, Dehaën enjoins redoubled attention, as a mistake is easily made; and again he asks himself what is its pathognomonic sign. Is it the pain? But nothing is more common than peripneumonia without pain. Is it the difficulty of respiration? But we observe this in violent fevers. Is it the softness of the pulse? No, for the pulse presents this softness only when the disease is far advanced and death is imminent. He then states that the pulse is developed in peripneumonia, but that it presents at the same time something wave-like and soft, and that this softness increases in proportion as the lung becomes less and less permeable to the blood, a smaller quantity of which reaches the aorta; and he finally arrives at this conclusion, which I transcribe literally: "*Sed tamen febris initio magna, respiratio multum læsa, tussis, anxietas, faciei rubor, urina cruda, peripneumoniam adesse concludere faciunt.*" (*Prælectiones pathologicæ*, vol. ii, p. 478.)

It is impossible not to admire the sagacity with which Dehaën discusses the value of the signs of these two affections, which the spirit of his time tended to confound and regard as only one. We recognize the experienced practitioner in the minuteness of the remarks which he makes on the pulse in pneumonia, remarks the truth of which daily experience still shows. But I ask, has he succeeded in solving the problem, and are the signs which he indicates sufficient to distinguish pleurisy, more than pneumonia? Such is the uncertainty in which have terminated the labors of so many celebrated pathologists! either to make of these two phlegmasiæ one disease, or to be unable to distinguish the one from the other, admitting, too, the possibility of their separate existence. Let us not be in haste, however, to throw aside the results of their studies and the fruits of their observations; let us rather inquire if it was possible for them to have done better, having as guides in their researches general symptoms only, and possessing no means of appreciating the physical signs which have since aided so much in the *localisation* of diseases of the chest.

At length, in 1808, Corvisart published a translation of Avenbrugger's *Treatise on Percussion*, with comments. The original work was printed at Vienna in 1763, and afterwards fell into total neglect, Stoll and Van Swieten being the only authors that mention it. The success of this mode of investigation has been constantly increasing since its revival, and it has now become the necessary companion of auscultation.

I find but little in this work relative to the diseases which we are considering. I scarcely think that Avenbrugger ever tried percussion of the chest in pneumonia or in pleurisy; it would have required a good deal of boldness and even temerity, at that period, to have added even the slight pain of percussion, to the pungent pain of pleurisy. Such trials are not generally made, except in cases where we know beforehand the result we seek, and rarely from mere curiosity; and hence all that Avenbrugger has accumulated in his very little treatise relates solely to chronic diseases of the chest, or of the heart. I shall avail myself of this in due time.

Before leaving this author, however, I will note the signs which he lays down for recognising dropsy of the chest: "Besides the general symptoms just enumerated (they are those indicated by the other authors), we observe that the affected side, if it be entirely full of water, is weaker and appears less moveable during inspiration; when it is percussed, no sound is elicited from any part, but if it be only half-filled with water a louder sound will be obtained in that part above the liquid. In this case the sound varies according to the position of the patient, so that it will follow the direction of the level of the liquid." (Op. cit., translated by Corvisart, p. 374). In these few lines, Avenbrugger makes an immense step in the diagnosis of effusions; and the reader will observe that his method indicates the height of the effusion, and the mode of ascertaining whether it be a liquid which fills the chest, or solid matter, such as the swelled lung; rendered impermeable to air; for in the latter case the change of position does not displace the seat of dulness.

Pinel was acquainted with the treatise of Avenbrugger when he wrote his *Nosographie philosophique*, and he has not neglected to apply this method to the diagnosis of the effusion which almost always accompanies pleurisy. He notices the dull sound on the side of the effusion; and he adds to it all the other symptoms which we have seen imputed by nosologists to dropsy of the chest. As to pneumonia and pleurisy without effusion (if there be any such thing), he distinguishes them by nearly the same symptoms which we have analyzed in Dehaën.

At last Laënnec appears, and the old question of the separation or the confusion of the two diseases is forever decided: for this purpose he begins by giving the precise anatomical characters belonging to each.

Inflammation of the pleura is always accompanied by an exhalation on the internal surface of the membrane. This exhalation, which Laënnec regards as a mode of suppuration peculiar to serous membranes, is present from the beginning of the inflammation, and results in the formation of false membranes and a sero-purulent effusion. False membranes form those whitish patches which cover the whole inflamed part of the pleura, and the external surface of the lungs. Sometimes more consistent, they form bands which pass from the lung to the pleura traversing the effused liquid. Susceptible of organisation, blood vessels may then be seen developing themselves in their interior, and they constitute those adhesions of the lungs to the ribs, so frequently observed in individuals who have suffered with pleurisies at remote periods.

As for the effusion, its nature varies extremely. Generally yellow, whitish, mixed with albuminous floculi which are only detached false membranes, it has been compared to whey. Its quantity is likewise subject to much variation: the more intense the inflammation, the more abundant are the false membranes, and we then find but a few ounces of effused serum; when the inflammation is less, and in feeble lymphatic subjects, false membranes are more rare, and the effusion may become very great and be confounded then with hydrothorax.

The parenchyma of the lung remains intact when the pleurisy is simple, save that it is pressed back against the mediastinum, and, in consequence of the compression which it experiences, becomes less crepitant than in the natural state.

Pneumonia presents in its anatomical characters, three well marked stages: *engorgement, hepatization and purulent infiltration.* An *engorged* lung is heavier, more compact, and less crepitant than in the healthy state; it retains the impression of the finger like an infiltrated limb; it is filled by a liquid which flows out abundantly on a section being made, mingled with globules of air which render it frothy; the red pulmonary tissue is then seen, retaining, however, its alveolar form.

The word *hepatization* indicates sufficiently well the condition of the lung in the second stage of inflammation; harder, heavier, no longer crepitating under the finger when pressed, it acquires a consistence analogous to that of the liver; internally it presents a very deep color varying from violet gray to blood-red; nothing flows out on making an incision, but by scraping the cut surface with the scalpel a sanguinolent serum is obtained, thicker and more turbid than that of engorgement; lastly, the pulmonary substance presents nothing cellular to the eye, but exhibits a granular appearance

2*

which is the peculiar anatomical character of pulmonary inflammation, and which exists only in this disease. Finally in the third stage, that of *purulent infiltration*, the pulmonary tissue is much less hard and consistent than in the second, and assumes a pale yellow color, analogous to that of straw. This color is owing to the infiltration of pus, which oozes out more or less abundantly when the lung is incised; the granular appearance of the parenchyma, which constitutes the essential character of the second stage, disappears in proportion as softening is effected. When the lung contains much black pulmonary matter, which is common in adults and old persons, the pus and infiltrated lung assume an ash-gray color, which many authors and especially M. Andral have noticed under the name of *gray softening* (*ramollissement gris*).

The reader will pardon me for having touched slightly on the anatomical characters of the two diseases under consideration; it was indispensable to the proper estimation of the difference existing between the two, and to show the great error of those who have considered them one and the same disease. Let us see now by what signs Laennec distinguishes each during life.

I will not here repeat the enumeration of general symptoms drawn from the fever, the pain and the nature of the expectoration; I have already discussed their value at sufficient length, in speaking of the nosologists who preceded Laennec, and I think I have demonstrated, that alone, they do not give to diagnosis the certainty which it should possess. Percussion of the chest produces a dulness nearly equal in the two phlegmasia, nor does this sign furnish infallible data for judging of the nature of the disease. Pretensions have been made to draw differential signs for each of these diseases from percussion, by the difference of the sounds obtained when the patient varies his position; but it was forgotten that in order for the liquid to be displaced by every movement of the patient, the chest should be partly empty, whereas it is always exactly filled by the organs which it contains. The only sign at all important furnished by percussion, is the extent of the flatness in pleurisy; in a short time a whole side of the chest becomes flat, whilst in pneumonia, the chest loses its sonorousness by degrees, and the flatness remains for a long time circumscribed. But let us turn to the results furnished by auscultation.

*In pleurisy*, a liquid being interposed between the parietes of the chest and the lung, 1st, the respiratory murmur ceases to be heard over the whole of the affected side, unless, however, it be in an extent of about three fingers' breadth along the vertebral column (in which place the lung is found, compressed on all sides by the effused liquid); 2d, if the patient be made to speak, the voice assumes a peculiar trembling character, which Laennec has named *egophony*.

*In pneumonia*, 1st, the essential sign from the beginning is *crepitation*; it occurs in no other affection than hemoptysical engorgement (which is known, indeed, to be a real species of pneumonia, of which I will speak further on, under the name of lobular pneumonia); 2d, when the second stage of pneumonia commences, the crepitant râle disappears, to be replaced by a bronchial respiration, which proves that the air does not penetrate into the pulmonary vesicles, but only into the bronchiæ, the parietes of which are rendered thicker by the hepatized parenchyma; 3d, when the patient speaks, the voice reaches the ear with a very strong resonance, without trembling, but not so directly as in pectoriloquy; 4th, when suppurative softening takes place, the mucous râle is heard.

Finally, *in pleuro-pneumonia*, the combination of the symptoms of the two diseases will prove that the phlegmasia involves at the same time both the pleura and the lung.

The different abnormal sounds which I have just indicated, are not always very distinct, and often it is not without difficulty that some of them are distinguished from others. Thus, in pleurisy, it is not always easy to perceive egophony and to distinguish it from bronchophony; each, in fact, consists in a resonance of the voice, through the parietes of the thorax, which, much less direct and much less distinct than in pectoriloquy, is sometimes equally strong in both. A similar cause indeed produces them, viz: thickening of the walls of the bronchiæ, which in bronchophony arises from hepatization of the lung, and in egophony from effusion; the only difference consists in the trembling character given to egophony by the fluid which the voice traverses in passing to the ear of the observer. This character is the more easily detected, as one becomes more practised in auscultation. But in the most embarrassing cases, and when there is the greatest difficulty to determine which it is that is heard, error may be prevented by reflecting: 1st, that bronchophony is never heard but in a circumscribed space, whilst egophony is heard over the whole, or at least the greater part of one side of the chest, a space which the former could not occupy without the severity of the general symptoms presented by the patient evincing that it was not a simple pleurisy that affected him; 2d, that the seat of bronchophony is invariable, whatever be the position of the patient, whilst, when egophony occupies a part only of one side of the thorax, which in that case is always the most dependent, it can be displaced by a change of position; 3d, and finally, that it is impossible to hear at the same time, and in the same zone of the lung, the natural respiratory murmur and bronchophony, because if the pulmonary vesicles be still permeable to air, the parenchyma no longer presents that indurated condition necessary for the voice to resound in the bronchiæ as in tubes having solid walls. Now, in cases where egophony is perceived most distinctly and over the most extensive surface, the respiratory murmur is always heard at the same time in a space of about three fingers breadth along the vertebral column, the lung being forced back there by the effusion.

When the effusion is considerable, egophony disappears, but the dullness increases, the affected side of the chest is dilated, and this dilatation is often perceptible to the eye; sometimes the diaphragm is pushed down by the effusion, producing a considerable prominence of the abdomen, which cannot be touched without increasing the oppression; lastly, the persistence of the respiratory murmur along the spine will guard against all error. When the effusion diminishes, the egophony reappears; in which case it is always at the superior part of the thorax that it is first heard; this might be accounted for by saying that it is more perceptible at the surface of the effused liquid. But the better reason is, that the lung, becoming more easily dilated in proportion as the effusion diminishes, is always found separated at its summit from the walls of the thorax by a thinner layer of liquid than at its base. Nevertheless it should be stated that this returning egophony is not always heard, as when long-standing membranous bands hinder the lung from resuming the degree of expansion which is natural to it in the healthy state.

Pleurisy may be double: in this case percussion affords no information, any more than the inspection of the chest. But the complete absence of the respiratory murmur in both sides of the thorax, except along the spinal column, would prevent any mistake, if for the most part the formation of an effusion in the side previously healthy, were not a phenomenon of the state immediately preceding death.

Finally, in those very rare cases where pleurisy exists without effusion, it will be recognized by the grating sound (*bruit de frottement*) produced by the false membranes lining the pleura and the corresponding part of the lung. And as there is always pneumonia at the same time, the symptoms of the latter will be found constantly predominating over those of pleurisy.

The analysis of the stethoscopic signs of pneumonia will not exhibit fewer proofs of their absolute certainty than those of pleurisy have presented.

I have said that the essential sign of pneumonia was the *crepitant* rhonchus, the name of which was suggested by its resemblance to the noise made by salt, which decrepitates when submitted to a moderate heat. This rhonchus is heard in pneumonia only (it matters not whether general or circumscribed); it is therefore justly regarded as its pathognomonic sign. It occurs in the pulmonary cellules, which are filled with a liquid, but are still traversed by the air. M. Andral, from consideration of the fact, which is incontrovertible, that the crepitant rattle results from a mingling of air and liquid, observes that the mucous rhonchus and gurgling are produced by the same cause. As a proof of it, there is a certain number of cases in which it requires a very practised ear to distinguish crepitation from the mucous rhonchus; and a very great number in which it is extremely difficult to distinguish the latter from gurgling. Reasoning thus by analogy, M. Andral concludes that these different sounds, arising from an identical cause, present differences in consequence solely of the size of the cavity in which they occur. Thus, gurgling is heard in great excavations, the mucous rattle in the large bronchiæ, and the crepitant rhonchus in the extremities of the latter, and especially in the pulmonary vesicles. (Andral, *Clinique Médicale*, vol. 1, p. 524). These observations of M. Andral are very just, and clearly establish the seat of crepitation. But in the production of this phenomenon, one thing must be considered, besides the capacity of the place where it occurs: the nature of the liquid which fills the pulmonary vesicles plays a conspicuous part. Dr. Spittall, of Edinburgh, author of a Treatise on Auscultation, has made an experiment which proves that the less viscid this matter is, the more distinct is the crepitant rattle. He put equal quantities of liquids differing in density and tenacity into bottles, and, after agitating them, applied them in succession to the ear to hear the crepitation produced by the bubbles of air which rose and burst on the surface. The degrees of crepitation furnished by this means, differed with different liquids, and those which produced a crepitation the most resembling that of pneumonia, were *serum* and urine. These experiments prove that, in the onset of pneumonia, it is not the matter expectorated which fills the pulmonary vesicles and the extremities of the bronchiæ, since the more distinct the crepitation is, the more serous is the liquid which fills the vesicles. Now, the expectoration of pneumonia being always viscid, it must necessarily be produced by the upper bronchiæ and throat.

Crepitation is heard in the engorged points only: it therefore furnishes an infallible means of ascertaining, within a few lines, the whole extent of the engorgement, and of telling with an almost mathematical precision what part of the lung is attacked, and what part is still healthy. What means of diagnosis had ever furnished such precision as this?

When pneumonia passes from the first to the second stage, that is, when hepatization succeeds engorgement, the crepitant rale disappears; but then the flatness on percussion, the absence of the respiratory murmur in a circumscribed point of the thorax, and the existence of bronchophony in the same point allow no hesitation concerning the nature of the complaint, especially when added to a viscid and bloody expectoration, which is so peculiar to pneumonia that Laënnec proposed to name it *pneumonic*. I will not repeat what I have said above, of the means of distinguishing bronchophony from egophony: its principal characters are known to be its existence in a point ordinarily circumscribed, which does not change with the position of the patient, and its want of trembling. Let us add that, in those parts of the lung not invaded by inflammation, it ceases to be heard, and the respiratory murmur is more clearly perceived the greater the distance from the hepatized point.

The resolution of pneumonia, after it has reached the stage of hepatization, is announced not merely by the diminution of the general symptoms and the change in the nature of the expectoration, which becomes more and more white or yellowish, and in which blood is less and less perceptible, but also by the return of the crepitant rale, mingled with the murmur of pulmonary expansion, which becomes more marked from day to day until it finally exists alone. The return of crepitation in the decline of pneumonia, and of egophony in the diminution of a pleuritic effusion, have the great advantage of affording the practitioner the material proof of the success of his treatment, and of making it impossible that he should be misled by a deceptive mildness and amendment in the symptoms, such as is sometimes seen immediately preceding the catastrophe. At the same time, the observant physician finds in them a proof that our diseases abate by returning through the same stages which they presented whilst advancing; and that an engorged organ, undergoing resolution, does not differ physically from what it was when the engorgement was in progress, only that the vital force acts in an inverse mode: in the onset, by accumulating disorganizing materials; and in the decline, by taking up those materials again by means of absorption, leaving the organ in its primitive and normal state.

Under the name of *lobular pneumonia* a particular species of inflammation of the lung has been described, in which the engorgement is limited to one or more central nuclei which sometimes scarcely attain the size of a walnut. In this case is it always easy to discover crepitation? Authors are divided on this point. Laennec affirms that he always found it, and it is certain that the more experienced one is in the art of auscultation, the more rare do difficult cases become; and the art, therefore, should not be made responsible for the unskilfulness of those who practise it. In the case of which we are speaking, there is a combination of crepitation, which is heard in a deep and circumscribed spot, with the respiratory murmur, heard more superficially, but with its normal type and only a little more rude or puerile. When lobular pneumonia passes to the second stage, there is likewise a simultaneous existence of the respiratory murmur and bronchophony. If the central pneumonia increase and extend towards the surface of tbe lung, the respiratory murmur diminishes more and more, whilst bronchophony becomes nearer. And it is well to remark, that lobular pneumonia is a disease in which an error in diagnosis offers the least danger; for it is not a serious affection, and becomes so only when it has a tendency to invade considerable portions of the lung, and then it will be easily recognised by the stethoscopic signs already pointed out.

There is an affection, but little studied before the time of Laennec, the stethoscopic signs of which are slightly blended with those of pneumonia, and consequently it is sometimes difficult to be distinguished, the more so as it frequently follows the latter disease. I mean *œdema of the lung.* The results of auscultation in this affection, are a diminution of the respiratory murmur, and a crepitant rhonchus the formation of which is naturally explained by the infiltrated state of the lung, which nearly approaches what we have described under the name of *engorgement.* But in *œdema,* the crepitant rale is much more humid than in pneumonia, for which reason it has been called *sub-crepitant.* Then, the general symptoms are not the same: the flatness is generally less than in pneumonia, because the lungs always contain air mixed with serosity, which does not happen in pneumonia; the expectoration is more abundant than that of the latter disease and differs from it essentially, as it is completely colorless, analogous in aspect and consistence to the white of egg beat up with water. Lastly, though the dyspnœa is generally so great as often to simulate that of asthma, which is owing to both lungs being generally infiltrated at the same time, still the symptoms of reaction are wanting, there is no fever and but slight cough, which ought necessarily to preclude the idea of pneumonia.

Such is the new symptomatology which Laennec has not substituted for the old, but added to render it complete and supply its deficiencies. It will now be easy for me, after this exposition, to enumerate the improvements which it has caused in the art of diagnosis.

Thus, 1st, auscultation has at length decided this great question, which pathological anatomy alone had not been able to solve; whether pleurisy and pneumonia are or are not two distinct diseases. And let it not be said that this distinction is useless to therapeutics! for who will dare say that an acute pleurisy should be treated absolutely in the same manner as an acute pneumonia? Would not the difference in seat necessarily lead to a modification in the therapeutic means? Who does not call to mind this essentially practical distinction which has been made between local and general sanguineous evacuations, accordingly as they are made for the phlegmasia of a membrane or for that of a parenchyma?—that in the latter, venesection alone is capable of effecting a diminution of the hyperemia of which a parenchymatous organ is the seat; whilst in the phlegmasia of serous membranes, bleeding from the capillaries has a much more prompt and certain derivative action. When after the acute stage has passed, and revulsives must be thought of, will it not be of the highest utility to know the precise seat of the disease, so as to decide upon the proper place for their application? will not the indications vary according to the aspect which the disease may take? and will a pleuritic effusion be treated by the same remedies that we employ in a pneumonia, the resolution of which is accomplished slowly? All these things are so evident that there is no need of further remarks to make them understood.

2d. In enabling the progress of the two diseases we are considering, whether favorable or otherwise, to be followed, auscultation has rendered an immense service to the practitioner, who can thus judge of the effect of the remedies that he employs. I have already said, that the general symptoms are often deceptive, and sometimes have an apparent mildness which induces a security fatal to the patient. But auscultation properly applied, nearly assimilates the treatment of pleurisy and pneumonia to that of a wound, the daily modifications of which can be ascertained by the eye or the fingers of the surgeon.

3d. Auscultation, by enabling the precise seat of pneumonia to be determined in all cases, and by showing, within a few lines, the extent of the parenchyma which it invades, has disclosed this curious fact, which pathological anatomy alone would not have shown so well, nor so completely, that pneumonia generally begins at the base of the lungs, that it rarely commences at the summit and then is of a more serious character, as it is almost certain to involve the whole lung. And this observation, curious in itself, has furnished a very powerful argument to the opponents of that erroneous theory, which makes pulmonary phthisis a consequence of chronic pneumonia. In fact, tubercles are always developed in the first instance at the summit of the lung, as we shall see in treating of that disease, whilst pneumonia always begins at the base. Does not this difference alone prove already that the nature of the two diseases is not identical?

4th. In pointing out the physical signs of effusion, an almost constant result of pleurisy, Laënnec has rescued surgeons from all chance of error hereafter in the operation for empyema; for it is evident, that, whatever be the nature of the effused liquid, serum, pus, or blood, the signs which indicate the presence of a fluid in the cavity of the chest do not vary. It is fearful to think how uncertain, before the discovery of auscultation, were the symptoms from which was deduced the indication for thrusting the bistoury through the parietes of the chest. In the patient operated on by Morand, and whose history he has inserted in vol. ii. of the Memoirs of the Royal Academy of Surgery (p. 545), this celebrated surgeon declared the necessity of empyema from the following symptoms only: fever, insomnia, pains in the head, in the neck, and in the whole epigastric region, principally in the left hypochondrium and in the chest of the same side; œdematous swelling of the whole left side of the body, suffocation to such a degree that the patient could scarcely move, or even spit or speak; impossibility of resting otherwise than on the back, *slightly inclined forward* (Morand doubtless intended to say *seated and sitting up in bed*); frequent syncope. Morand, assisted by Moreau and Louis, who, it appears, were not so well convinced as himself of the presence of pus in the thorax, made an exploring puncture at the place selected on the left side; and the issue of a stream of serum confirmed the opinion which he had formed. But in thus deciding the nature of the complaint, was Morand guided by the mere analysis of symptoms only? Did he not evince that species of presumption so natural to a superior man who trusts to his genius? He guessed well; but how many have been deceived! and how often has a useless and therefore dangerous operation been performed on a chest without effusion! We read in Dionis, that a surgeon who was otherwise skilful, operated for empyema on the Duke de Mortemart, and found nothing in the chest. "A circumstance nearly similar to this," adds Dionis, "happened at Versailles in 1703, to one of the surgeons of the king. M. Helvetius came to see one Batteville, upholsterer to the king, who had been ill for a long time, and was complaining of a pain in the right hypochondrium; having touched the spot, he thought there was matter, and advised the surgeon to make an opening, which he instantly did; nothing followed the incision, and the patient died of the operation." (Cours d' opérations de chirurgie, by Dionis, vol 1, p. 435).

The contrary error is more frequent, and though less pernicious in itself, it is always injurious. In fact, examples are common of patients who have been found on dissection to have enormous pleuritic effusions, the existence of which had not been suspected during life. Professor Boyer cites from Ledran a remarkable example: "A patient was brought to La Charité, in whom, two days before, a deep abscess under the angle of the right maxillary bone had been opened. The third day the suppuration was suppressed, the patient had a chill, and felt a smart pain in the left side of the chest, with considerable oppression. The great symptoms or rather signs which denoted that suppuration was going on, lasted three days, after which the patient was infinitely better. The only thing of which he complained was a sense of fluctuation within the chest whenever he moved, and a feeling of oppression when seated. He laid equally well on both sides and had no other sign of an effusion than this fluctuation which he alone perceived. . . . . . Ledran, thinking the case equivocal, took the advice of several colleagues, a majority of whom decided to wait for something more certain before operating. The fever continued and was accompanied with cold sweats, and the patient died on the eighth day. On opening the body, there were found about five pints of pus effused in the chest." (Boyer, Traité des Maladies Chirurgicales, vol vii, p. 362). If auscultation had been practiced in this case, together with percussion of the chest, I ask, would the uncertainty of Ledran and his colleagues have been possible? "I am persuaded," says Laënnec, "that the operation for empyema will become more common and more useful, in proportion as mediate auscultation shall come into general use. In fact, up to this time, simple empyema and idiopathic hydrothorax, have scarcely ever been recognised, save in cases where the disease was of long standing, or of an aggravated character; yet many cases presenting even these conditions escape the attention of the most skilful surgeons and physicians, and for a much greater reason do the less severe cases which would offer more hope of saving the patient." (Traité de l' Auscultation médiate, vol. ii, p. 219).

5th. Another improvement to be added to those which auscultation has made in the art of diagnosis is this: henceforth to the attentive physician, there will be no more of those latent pleurisies, of which the ancients have reported so many cases, and which for the most part they appear disposed to regard as the result of the purulent melting of the lung. The stethoscope will always disclose the commencement and progress of the effusion, at the same time that it will furnish no less certain information relative to the organic lesion of the lungs, the symptoms of which predominate and mask those of the pleurisy.

6th. Lastly, with an attentive observer, there cannot be a development of pneumonia, the symptoms of which remain unknown, any more than there can be a latent pleurisy. So soon as the pulmonary substance becomes engorged, crepitation is developed; and it is by means of this sign that M. Piorry has been enabled to ascertain that the sanguineous and serous engorgement, almost always met with in the posterior part of the lungs of dead bodies, and which was regarded as a cadaveric phenomenon, a mechanical result of the gravitation of the fluids, often precedes death a good while, and constitutes a real disease mistaken for bronchitis; and that to this disease, which he has described under the name of *hypostatic pneumonia*, is to be ascribed the death of a great number of patients. We must not be deceived, and attribute to this phenomenon of the latter periods of life, a more important part than it really plays. It is certain that when death approaches, as the consequence of any disease whatever, in proportion as the force of the vital principle relaxes, the organs previously sound no longer oppose a sufficient resistance to the causes of destruction, against which they are in an incessant struggle during life; and a species of vital decomposition precedes by some days the fatal moment when the body shall be unreservedly subjected to the exclusive influence of the physical agents which surround it, and of the chemical elements which compose it. Nevertheless it still follows from the interesting researches of M. Piorry, that there is often an inflammatory reaction in the dependent portions of the lung, whenever the weight or some mechanical impediment causes the fluids to remain there. Thence the indication to prevent the patient from remaining constantly on his back, and to make him lie alternately first on one side, and then on the other; and every time that the nature of the disease does not oppose it, to make him get up a while, and if he is able, walk about.

### CHAPTER II.—OF PULMONARY CATARRH AND PHTHISIS.

I purposely include these two diseases in the same chapter, for before Laennec their confusion was almost inevitable. Indeed, if we consider these two affections in their extreme and most distant points, there is a very wide difference between them; and between a slight cold of a few days and a phthisis arrived at that state called by Bayle the third stage, error is impossible, and there is no need of the stethoscope to guard against it. But in certain chronic catarrhs accompanied by an abundant expectoration, and which last sometimes for whole years, how difficult it would be, without the stethoscope, to decide whether or not there be phthisis.

Cullen, endeavoring in his *Practical Medicine*, under the article *pulmonary phthisis* (vol. ii, p. 159), to establish a differential diagnosis between this disease and catarrh, says: "Catarrh or the expectoration of mucus is often accompanied by fever; but this fever is never, so far as I have been able to observe, like that which I am about to describe as hectic fever. This latter is, in my opinion, the most certain mark of a purulent state of some part of the body." He then describes hectic fever, to which he assigns as an essential character, its daily recurrence, chiefly at noon and in the evening.—There is a remission in the morning, but rarely apyrexia. It is accompanied with night sweats, and the urine deposits a furfuraceous brick-colored sediment. But similar fevers are sometimes observed in chronic catarrhs, which gradually undermine the constitution and sooner or later cause death, without there being the slightest ulceration, or the least tubercle in the pulmonary parenchyma. This is frequently observed in old persons, or in young children who die of pulmonary catarrh, following hooping cough.

Besides, we find this character of hectic fever indicated by almost all authors as the specific character of phthisis. Sauvages, who places this disease in the class *cachexiæ* and order *emaciations*, has thereby shown what idea he had of it. Shall I speak of the distinction that he makes between *tabid* fever, the character of which chiefly consists in emaciation, and *hectic* fever which takes its character from the intensity of the fever? This is too subtle a distinction. So with the twenty different species of phthisis which he describes, or rather points out; for in following the indications furnished by him it would be difficult to establish a certain diagnosis: and as to the differential diagnosis between phthisis and pulmonary catarrh, we ought still less to ask it of him; for it is pathological anatomy alone which has pointed out the material differences which separate these two diseases, and nowhere in Sauvages is pathological anatomy referred to.

We should not, however, believe that pathological anatomy alone, however well studied, could have brought about that immense progress which the stethoscope has made in the art of diagnosis of the diseases of the chest. What author has been more engaged in pathological anatomy than Portal, and yet what work is more barren of facts calculated to advance the science than his, entitled: *Observations sur la nature et le traitement de la phthisie pulmonaire?* This work is composed of about one hundred and eleven observations on pulmonary phthisis, of which I find forty that terminated favorably. This result alone, already suffices to excite distrust in one who knows that, unfortunately, the most enlightened therapeutics have not yet been able to cure forty out of one hundred and eleven phthisical patients. We soon have the key to his success; when we see the author exclaim; "How many simple colds run into pneumonia or pulmonary phthisis in consequence of a stimulating treatment!" (Op. cit. vol. i, p. 369). In general Portal gives the name of phthisical to every patient, coughing, spitting, wasting and having a hectic fever. I have already said that we frequently find persons having only chronic pulmonary catarrhs, who likewise present a fever with emaciation, and yet the anatomical difference between catarrh and phthisis is so important that we may, without asking too much, demand of the authors who have written on these diseases, symptoms which distinguish the one from the other. In catarrh there is only a hypersecretion of mucus in the inflamed bronchiæ, in phthisis there is an organic alteration of the lung.

Although having the scalpel constantly in his hand, and terminating all his cases of phthisis by the autopsy of the subject, it would seem that Portal had no precise idea of the nature of the organic alteration which essentially constitutes pulmonary phthisis. Scarcely anywhere does he speak of tubercles, but of grumous concretions, of abscesses scattered in different parts of the lung. In the case of the abbé de Lamothe (op. cit. vol. i, p. 175), he gives as an example of phthisis from plethora, the history of a true pleuro-pneumonia, which the details of the autopsy do not permit us to mistake. But what still more confirms the judgement which I have passed on this author, is the fact that he establishes as many species of phthisis as there are causes capable of producing this degeneration of the pulmonary parenchyma. He describes *scrofulous, hereditary and plethoric* phthisis; phthisis *the consequence*

*of inflammatory diseases of the lung* (under this name we find described true chronic pleurisies); and phthisis following *exanthematous, catarrhal, rheumatic, scorbutic, calculous and venereal fevers.* Certainly the study of causes is not to be despised, any more than that of general and constitutional diseases with which phthisis may be complicated; but is it a sufficient reason for establishing all these different species, which, at the bottom, differ in nothing from each other?

However, I will not leave the work of Portal without awarding him due praise for having recognised the true nature of vomicæ, those abscesses which have attracted so much attention from nosologists. Portal regards them as being produced by an agglomeration of tubercles: "Vomica is on a large scale," says he, "what tubercle is on a small one." (Op. cit., vol. ii, p. 211). We shall see that this idea has been fully confirmed by the researches of Laennec.

Thomas Reid, author of an English treatise on pulmonary phthisis, does not better distinguish this disease from simple catarrh; the very definition which he gives, proves that he had not a very clear idea of its nature. "Pulmonary phthisis, when fully confirmed," he says, "may be defined, the expectoration of a purulent matter from the lungs, by the repeated efforts of a painful cough, accompanied by a fever of a peculiar kind, which produces morning sweats, suffers remission in the afternoon, and soon causes a considerable loss of strength and flesh." (*Essay on the nature and treatment of pulmonary phthisis*, translated by Dumas, p. 4). We find nothing in this definition that specially characterises phthisis, nothing which stamps it with a particular seal, distinguishing it from other diseases with which the lung may be affected, and above all from chronic catarrh, with which it is most likely to be confounded. Are there not indeed catarrhs to which this definition, or rather description, would be perfectly applicable in all points, without excepting the fever and loss of strength and flesh? Not that we do not see in Reid a very manifest design to distinguish real and consequently incurable phthisis (for, according to him, there is no cure to be hoped for), from the diseases which simulate it. "The numerous cures of confirmed phthisis which are cited," says he, "often deceive us, and gain credence by our mistaking mucus for pus." (Op. cit., p. 39). Wishing to prevent similar errors, he points out the characters by which pus is distinguished from mucus. The chief of these consists in the fact that pus mingled with water separates on standing, and is precipitated to the bottom of the vessel, whilst this does not happen with mucus. But this differential character, to which Reid attached so much importance, is altogether insufficient to distinguish pus from mucus. Besides what would it prove, even were it as infallible as it is unfounded? What more common than puriform sputa in pulmonary catarrh? "We should give but little confidence to the inspection of the sputa in pulmonary phthisis," says Laennec, "because even the most characteristic, such as those which are ash-colored, or puriform and vermicular, are frequently met with in chronic catarrhs; and, moreover, the expectoration of consumptives is, excepting a thousandth part, nothing but the product of a pulmonary catarrh, which almost always accompanies the tubercular affection of the lungs. (Traité de l' Auscultation médiate, vol. i, p. 689.)

If the efforts which Reid has made to distinguish affections purely catarrhal from true pulmonary phthisis have not been crowned with success, we must still praise him for having a clear idea of the essential difference between the two diseases. Phthisis is always the result of tubercles in the lungs, a good description of which he borrows from Stark. (Reid, op. cit,, p. 46). The parenchymatous substance of the lungs compressed by these foreign bodies, and removed, so to speak, from the vital influence, thus becomes susceptible of being dissolved and converted into pus, which of itself, by its own solvent properties, leads to softening of the tubercles, and causes them to burst into the interior of the bronchial ramifications. (Reid, op. cit., p. 52). All these things are perfectly correct, and, in truth, little has been added to this theory. We shall not, perhaps, find the same correctness in Reid's explanation of the hectic fever, which always accompanies the destruction of the lung, and which he states depends on the fact that this organ, no longer receiving the same quantity of air as in the normal state, does not exhale the same proportion of lymphatic fluid. The portion of this fluid thus retained in the body, combines with the superabundant phlogiston to produce the fever. (Reid, op. cit., p. 107.) It was really not worth his while to take so much pains in combatting the ancient notions which attributed the fever of phthisis to the absorption of pus from the lungs, and its mixture with the blood, in order to substitute for it a theory so poor, so improbable, and so inferior to that which he rejects. Be this as it may, Reid can be said to have advanced farther, relative to the point which we are discussing, that is, the distinction between phthisis and pulmonary catarrh, than his translator, Professor Dumas of Montpelier. The former has at least tried to establish a differential diagnosis, and the insufficiency of his means of investigation is the only cause of his failure. Dumas, on the contrary, in the preliminary discourse which he has prefixed to his translation of Reid, endeavoring to prove that many chronic diseases take their origin in the pituitous constitution of the humors, adds: "that catarrhal fevers have a close relation, a manifest filiation with the disease of the chest called pulmonary phthisis" (Reid, op. cit., *Disc. prelim.* of Dumas, p. 16); and further on: "not only does catarrh give rise to phthisis, but the catarrhal fevers have also a great affinity with pulmonary hectic fever" (p. 67). Not wishing, though, to judge the ideas of Professor Dumas from a few passages taken from his preliminary discourse, I leave this subject, and pass on to Baumes, another professor of the University of Montpelier, so fruitful in illustrious men.

Baumes, author of a treatise on phthisis pulmonalis, which in 1784 took the prize of the ancient Royal Society of Medicine, lays down ulceration of the lung as the essential character of pulmonary phthisis, in the same manner that ulceration of the liver, mesentery, or kidneys constitutes hepatic, mesenteric or renal phthisis. Thus, then, phthisis pulmonalis is, according to him, an emaciation caused by the purulent liquefaction of the lungs. Beaumes was fully aware how much this definition restricted the cases of real phthisis, and appears to have seen that it was obnoxious on account of its narrowness; for he immediately adds: "But the non-ulcerative affections of these same organs which, by their influence on the animal economy, bring about the destruction of the machine and death, are doubtless worthy of a particular denomination: it is thus that non-suppurating tubercles should constitute a special disease, to be called *pulmonary hectisis* (*étisie pulmonaire*), as schirrous engorgement of the mesentery, of the liver, or of the spleen, constitutes mesenteric, hepatic or splenitic *hectisis* (*étisie*), etc. (Op. cit., vol. i, p. 8)". In thus creating two genera of diseases of the chest, both having for their essential character emaciation and wasting, Baumes was ignorant of what has since been so well established, first by Laennec and then by Andral, Louis, and other pathologists who have followed in the same path, that these two genera, so distinct in his work, are confounded together in nature, and that the one always precedes the other; for pulmonary ulceration is always the result of the existence of tubercles which, after having remained for a longer or shorter time stationary and in a dry state, soften, and dissolve to some extent in the pus formed in consequence of their presence in the pulmonary parenchyma, and, being eliminated with it, leave an ulceration in the place they occupied. What line of demarcation, then, can be drawn between these two states? By what differential signs will they be recognised? It is still by an attentive examination of the products of the expectoration, that Baumes expects to find the distinguishing marks: and like Reid he multiplies experiments in seeking to distinguish pus from simple mucus. I will not here detail all the minute processes which he employs to attain this end, I will not cite all the reagents to whose action he submits the products the nature of which he wishes to determine; they can be seen in his work (vol i, p. 66). Were these processes as infallible as they are unfounded, what would they prove, since simple catarrhs, without ulceration of the mucous membrane of the bronchiæ, are sometimes accompanied by sputa differing in nothing from those of confirmed phthisis?

Although not regarding tubercular phthisis, as a real phthisis, unless when the tubercles have passed to the stage of softening, Baumes could not do otherwise than indicate the signs of the presence of dry tubercles in the lungs. He does so, after having described the general characters of the temperament which, according to him, predisposes to tubercles, and which is only the scrofulous temperament exaggerated. The pathognomonic signs which he indicates are: embarrassed respiration, which would induce the belief of asthma; a continual dry cough, increasing in frequency and intensity, in proportion as the tubercles become more numerous; difficulty of making full inspirations, which are followed by cough; short breath from the slightest fatigue; impossibility of singing; febrile pulse; emaciation; irregular sweats; general pallor, the cheeks alone remaining colored; suppression of the catamenia in women. These symptoms are correct, but at the present day they only warrant us in suspecting tubercles, auscultation, combined with percussion, being alone capable of rendering their presence certain. But in Baumes' view, this group of symptoms characterises only the predisposition to phthisis; and he was so completely ignorant of the inevitable necessity of the softening of tubercles, that he afterwards devotes some lengthy pages to the study of the proper means for encouraging a resolution of them, and for preventing hectic fever, without which he cannot conceive of phthisis. We shall again see that to have proved that tubercles once formed never retrograde, and can only be eliminated after having gone through the periods of softening and suppuration, is another of the triumphs due to auscultation. On the subject of vomicæ, how much less advanced is Baumes than Portal, who, however, wrote long before him! We have seen the latter acknowledge the identical nature of vomica and tubercle, the only difference being in their volume. According to Baumes, a vomica may be of two different species: inflammatory, or lymphatic. In both cases, it has nothing in common with phthisis; and the reason that many authors think they have cured consumption, is because they have treated patients having vomicæ only. (Baumes, op. cit., vol. i, p. 408).

But if so many authors have been deceived, error must indeed be easy; and then, how is it to be avoided? This is what Baumes does not tell us.

Passing finally to the description of unequivocal ulcerated phthisis, Baumes indicates the following as the principal symptoms: hæmoptysis in the beginning, pains in the chest, sore throat and frequent hoarseness; a cough, somewhat resembling hiccup, and frequently causing only a sudden shock immediately before expectoration; change in the color of the face and flushing of the cheeks; frequent vomiting, by no means in proportion to the violence of the cough or the quantity of ingested aliments; night sweats, seldom involving the whole surface. Lastly come the signs drawn from an examination of the sputa, and from the presence of hectic fever. (Baumes, op. cit., vol. i, p. 59). In this description of open phthisis, we recognise the judicious and enlightened observer, who suffers nothing to escape him, and knows how to appreciate the importance of the most minute detail. Unquestionably this picture has an air of truth that would not allow of any indecision; but how many cases of manifest phthisis in which we find no such assemblage of symptoms! For example, how many patients are there whose lungs contain even considerable tubercular caverns, without hectic fever!

I find but little to notice in Bayle, although he has published a volume on the subject under consideration, (Recherches sur la phthisie pulmonaire, 1 vol.), in which there are some excellent anatomical details in reference to this disease. To this author is due the division, so long admitted, of the symptoms of phthisis into four stages or periods: *incubation*, *increase* (*début*), *confirmation* and *suppuration;* but he does not indicate the precise limits of each of them, nor any method of distinguishing the passage of one into the other, still less how to ascertain whether there is simple catarrh, or organic alteration of the lung: and, in fact, he has not been able to solve this problem better than the other authors whom I have cited, on account of the insufficiency of the signs upon which he was obliged to found a diagnosis.

But suppose it were otherwise, that these diagnostic signs were of sufficient value and importance to enable us to affirm positively, whether or not there is organic alteration of the lungs, how could the unsound lung be designated in a case where one of them remained healthy? I very well know that in certain cases the thoracic pains of consumptive patients have such a fixed character, as to authorise the belief that the seat of pain is at the same time that of the disorganization. But with how many patients are these pains vague, often changing place, or if stationary, fixed between the shoulders, and hence attributed only to the fatigue and irritation of the bronchiæ, resulting from a continuance of the cough! I know well, also, that a knowledge of the precise seat of alteration is not immediately important, and will not modify, in the majority of cases, the therapeutic indications; but it matters not, this is a precision of diagnosis to which the use of the stethoscope has so much accustomed us, that at the present day an approximate (*à peu près*) diagnosis is not tolerated.

The method of Avenbrugger, suitably applied, enabled us to approach a little nearer this *localizing* diagnosis; but its author does not appear to have tried it in pulmonary phthisis. He speaks, however, of scirrhus of the lung and of vomicæ. Now, under these two names, we should understand infiltration of the parenchyma by tubercular matter, and then an abscess or cavity resulting from the sudden softening of the latter: for. as to real scirrhus of the lung, and degeneration of its substance into an evidently cancerous tissue, it is a rare thing, and probably would cause death even before the cancer could make much progress. Now, Avenbrugger indicates as a sign of scirrhus, the diminution or even suppression of all sonorousness in the affected part of the chest, at the same time that infrequent cough manifests itself, without expectoration, or attended only with viscid sputa, etc. (*Nouvelle Méthode pour reconnaître les maladies internes de la poitrine, par la percussion de cette cavité.* Translated by Corvisart, p. 275). Then describing the signs that denote the softening of the scirrhus, and that the pus by its accumulation is about to form a vomica, he points out this very important characteristic. "If you place the palm of your hand on the spot where the vomica has been discovered by percussion, whilst the patient expectorates, you will perfectly distinguish the noise of pus in the interior of the chest, and so while the patient coughs. . . . . . At this period, before the commencement of expectoration, the seat of the vomica recognised by percussion, yields exactly the sound of flesh when struck. But it yields an obtuse resonance as soon as the accumulated matter of a recently opened vomica is eliminated by the aid of a violent cough." (Ibid, p. 334).

The importance of all the physical signs mentioned here by Avenbrugger, it is needless to insist upon; their simple enunciation is enough to make us comprehend how much they have added to the precision of diagnosis. Suffice it to say that Laennec has in no wise changed them, but that auscultation has given them one degree more of certainty, by confirming them, or rectifying them when there was occasion, or lastly, by supplying their place in certain cases where it was impossible to obtain them. But I hasten to explain the changes which this method has brought about in the diagnosis of the two diseases in question.

In the first place, so soon as it has been practised with care, a differential diagnosis is at once indicated, although the labors of so many pathologists anterior to Laennec had not succeeded in establishing it; and this great result, the consequences of which are so important, is entirely due to auscultation. In pulmonary catarrh, the sonorousness of the chest being perfectly natural, we hear, in the part corresponding to the inflamed bronchia, a râle which varies in kind according to the nature of the catarrh; ordinarily sonorous, grave, sometimes sibilant, or else mucous when the expectoration is abundant. Besides, there is commonly an absence of the respiratory murmur in the part of the lung surrounding the diseased bronchia, a natural consequence of the obstruction of the latter by the mucus with which it is engorged. But this absence of the respiratory murmur could never be attributed to a pleuritic effusion, for its seat is too circumscribed for that; neither to a pneumonia, nor to tubercles still in the crude state, because it is not permanent, but may cease and reappear suddenly: a fit of coughing being sufficient to bring on expectoration of the matter which engorges the bronchia, thereby restoring the respiration to its primitive state, until a new engorgement causes it again to disappear. Moreover, in the three diseases just named, percussion of the chest produces flatness, whilst pulmonary catarrh is always attended with very great sonorousness. And it is thus that these two methods, combined together, afford a mutual support, and correcting each other, bring about results of almost absolute certainty.

If the pathognomonic signs of pulmonary catarrh are so simple and easily recognised, those of phthisis are not less distinct.

Tubercles first accumulate at the summit of the lung, and it is almost always below the clavicle that the first signs of them are discovered. It is to auscultation that we owe the knowledge of this fact. Not that, while yet very small, and separated from each other by a healthy parenchyma, they are revealed by the least physical sign; the health is still good, and if the patient has a cough, it is of so trifling a nature that he rarely thinks of consulting a physician. But when once these abnormal productions begin to accumulate, the sonorousness of the chest diminishes in exact proportion to their size and number; and if both lungs are affected, as they seldom are to the same extent, percussion beneath the clavicles will produce an inequality of resonance which will indicate the more diseased lung. In this way the right lung is known to be generally more often affected than the left, or at least to be almost always the first. At the same time and in the same points, the respiratory murmur ceases to be heard, and is replaced by a diffuse and more or less marked bronchophony. When the tubercles begin to soften, the cough is accompanied by a characteristic gurgling, and the respiration, becoming cavernous, denotes that an excavation is forming in the parenchyma. Bronchophony then gives place to pectoriloquy, which at first diffuse and obscure, becomes more and more clear and distinct. The dulness of the chest then disappears and is succeeded by a resonance which, at first might create the belief of an amendment, if the state of the patient, constantly growing worse, did not prove it to be apparent only. Pectoriloquy and cavernous respiration, which are true pathognomonic signs of pulmonary caverns, facilitate diagnosis to such a degree that not only the situation of the cavern, but likewise its dimensions can be exactly determined. These results have been attained by all persons a little practised in the art of auscultation. But if the cavity be superficial and very near the surface of the lungs, so that a thin layer of parenchyma separates it from the cavity of the pleura, percussion at the point where it is most superficial produces the sound of a cracked pot (*bruit de pot fêlé*), which is quite characteristic, and leaves no doubt concerning the cause that gives rise to it; and if a superficial excavation has a part of its thinnest wall not adherent to the pleura, it will be recognized by the muffled blowing sound (*souffle voilé*) which then accompanies the cavernous respiration and pectoriloquy.

Such was the importance of pectoriloquy, as a pathognomonic sign of the formation of a cavity in the pulmonary parenchyma following the softening of tubercles, that it was of the utmost consequence to determine its precise characters, and point out the causes capable of misleading the practitioner in the conclusions which he should draw from it. Valuable details on this subject may be found in Laennec, which I do not think it necessary to copy, as they are foreign to the question which I am treating; I think it right, however, to mention this fact, that the most evident pectoriloquy sometimes ceases to be heard for a longer or shorter time: a little mucus accumulated in the cavity which is its seat, or obstructing the bronchiæ which open into it, is sufficient for this. It is evident that in this case, the elimination of the matter by expectoration, is indispensable to the reestablishment of pectoriloquy.

In the appreciation of this phenomenon, there are some sources of error to be guarded against. Pectoriloquy being always produced by the resonance of the voice in the interior of an unnatural cavity, formed in the pulmonary parenchyma, the dilatation of the bronchiæ, which is sometimes equal to the size of the little finger when an entire bronchial ramification is dilated, or the volume of a filbert or even of a small walnut, when a part only of the bronchia is affected, must likewise produce pectoriloquy; and thus a mistake is easily made. M. Andral cites a case in which it was committed. (*Cliniq. Médic.*, vol. i, p. 187.) The 11th observation of M. Louis in his *Recherches anatomico-pathologiques sur la phthisie*, p. 235, is another example. But with some attention this error may in general be avoided. Dilatation of the bronchiæ, an ordinary result of chronic catarrh, is never preceded by hæmoptysis, is not accompanied by pains in the chest and symptoms of hectic fever, which are the ordinary concomitants of tubercular excavations. Moreover, in dilatation of the bronchiæ, pectoriloquy is never so distinct; it is generally heard over a space much too extensive to be attributable to a cavern, and lastly, it is almost always combined with sonorousness of the chest, which is scarcely compatible with the presence of tubercles in the lungs.

In general, pectoriloquy is the more clear and distinct as the cavern has denser and thinner walls, and as it is nearer the parietes of the chest: a cavern of middle size will afford a more evident pectoriloquy than a very small one. However, the phenomenon is sometimes very imperfect in large excavations. In those, for example, which attain a capacity equal to the volume of the fist, especially if there is a communication with some important bronchial branch, the pectoriloquy sometimes ceases altogether. The same is true in cases where rupture of the pulmonary parenchyma makes a communication between the cavity of the pleura and the excavation; but then pectoriloquy is replaced by two other phenomena equally certain: *amphoric respiration* and *metallic tinkling*. The amphoric respiration is rarely wanting, and much less frequently than the metallic tinkling, which requires for its production, that there should be but little liquid in the cavern and that this should in addition be filled with air communicating with the bronchia. I have said that pectoriloquy also ceases, when a tubercular excavation bursts and empties itself into the pleura: the symptoms of pneumo-thorax, complicated with pleuritic effusion, are then developed. M. Louis has remarked that this rupture is ordinarily accompanied by a feeling of acute and sudden pain, attended with dyspnœa, and followed by the development of the symptoms of acute pleurisy. (Op. cit., p. 475).

Such are the new signs which Laennec has given to facilitate the diagnosis of a disease so important and so grave as pulmonary phthisis. I might say, too, that to him belongs the honor of having demonstrated beyond controversy that vomicæ, those abscesses of the lung which gave rise to such diversity of opinion among the ancients, are but the result of the sudden softening and instantaneous expectoration of a mass of tubercles. We have already seen that Portal held the same opinion. Laennec, by confirming it, has rendered a true service to science. But as it was through his anatomical researches, and not by means of auscultation, that he arrived at this result, I ought not to dwell longer upon it.

Let us now see what improvement they have effected in nosology and therapeutics.

1st. A line of demarcation has been for ever drawn between pulmonary catarrh and phthisis. Differing from each other in their seat, which is the mucous membrane of the bronchiæ in the one case, and the cellular tissue of the parenchyma in the other; in their nature, which is an inflammation in the first, and in the second an accidental inorganic production, with which inflammation has nothing to do; in their prognosis, which is infinitely less grave in catarrh than in phthisis, they have been shown to differ further in their signs, which render it impossible that they should be confounded. Now, to be able to distinguish a phthisical patient from one who is not, a case in which the prognosis must be extremely unfavorable from one that offers many chances of cure, is felt to be important. The importance of this distinction would be greatly diminished, if phthisis pulmonalis could be engendered by degenerated pulmonary catarrh; but this opinion, generally received before the commencement of this century, has been shown to be entirely false. Although the wisely directed researches of pathological anatomy have contributed much to this result, auscultation has not been without its influence, and this is a second advantage to be noted.

2d. Indeed, carefully performed, and applied to a great number of patients, auscultation has shown that pulmonary catarrh does not contribute to the production of phthisis more than pneumonia itself. Persons laboring under pulmonary catarrh of many years standing, on being submitted to auscultation, have presented none of the signs which indicate the formation of tubercles; percussion of the chest eliciting a sound as sonorous as in the normal state, and the autopsy always confirming the diagnosis, and proving that pulmonary catarrh may extend its ravages even to ulceration of the bronchiæ, without a single tubercle being formed in the parenchyma. If phthisis is always ushered in by a cold, this is symptomatic only, and by no means the occasional cause of the tubercular eruption. What a difference there is, indeed, between the dry, hacking cough of phthisis at its commencement, and that of catarrh which soon takes on a humid character, in proportion as the expectoration becomes more abundant! Besides, nothing is more frequent than to find crude tubercles in the lungs of subjects that died of another disease, and had never had the slightest catarrh, and in whom these tubercles, still in a nascent state, had not been revealed by any symptom. I might add, indeed, that it is impossible to attribute tubercles to an accumulation of concrete pus, and that it is extremely probable that the physiological and morbid phenomena which we call *inflammation*, are not at all concerned in their production. But this would be entering into theoretical discussions foreign to my subject, and I forbear.

3d. One of the most interesting results which auscultation has afforded, is the constant existence of a pulmonary catarrh, latent or manifest, during the whole course of continued fevers. At the onset and often during the whole course of the fever, it is accompanied neither by cough nor expectoration, and would be completely unsuspected, if the expectoration did not give rise to a sonorous rhonchus, sometimes very loud. M. Louis even says, in his *Recherches sur la fièvre typhoïde* (vol. ii, p. 238), that this râle is ordinarily much louder, and occupies a much more extensive space than the ordinary acute pulmonary catarrh, and that such is its disproportion to the cough and dyspnœa, that it constitutes a special and characteristic mark for typhoid fever: "so that its presence in doubtful cases where the affection is slight and the cerebral symptoms little marked, might render the diagnosis clear." Be this as it may, Laennec, who first pointed out this important fact, adds: "That this catarrh is sometimes unmasked at the approach of the crises. It constitutes the crises by sputa, noticed by the ancient practitioners, and which I myself have frequently observed." (Auscult. méd., vol. i, p. 195).

4th. In children attacked with hooping-cough, auscultation gives all the signs which indicate the existence of a pulmonary catarrh, namely: a respiratory murmur weaker or even absent in some points, with good resonance on percussion, a puerile respiration in other points, and sometimes a mucous, sonorous or sibilant rattle. But all these signs disappear during the fits of coughing: so long as these last, nothing can be perceived but the vibration imparted to the trunk; the wheezing and prolonged inspiration, which then makes the pathognomonic character of hooping-cough, appears to take place entirely in the larynx and trachea. These results, agreeing pretty well with those furnished by pathological anatomy, leave it beyond a doubt that in hooping-cough there is sanguineous congestion of the bronchial mucous membrane. But is this sanguineous congestion the cause or effect of the disease? Although the expression of my opinion upon this point, is quite useless in the question which I am treating, since the reasons by which it is supported are not at all founded on the results of auscultation, I cannot forbear making it known. I will frankly state, then, that although auscultation combined with pathological anatomy has shown in hooping-cough the existence of an acute pulmonary catarrh, I can only see there a simple coincidence between bronchitis and the neurosis which constitutes this disease; a coincidence which is scarcely ever manifest, because the symptoms of the latter conceal those of the former. But sometimes the symptoms of each may show themselves separately, as M. Blache cites an example that occurred in one of his own children. In this young patient he observed a simple catarrhal cough, alternating with convulsive fits of the same. (Blache, art. Coqueluche, in the Dict. de Médicine, 2d edition). How, indeed, would a phlegmasia of the bronchiæ explain the peculiar and altogether abnormal course of hooping-cough; the absence of fever; the ease with which the spells of coughing return under the influence of moral causes, such as a fright or contradiction; the almost immediate return of the free exercise of the functions in the interval of the fits; the extraordinary obstinacy of this affection, far more difficult of cure than a bronchitis, etc., etc.?

In conclusion, I ought to say that Laennec, at the time of pointing out the existence of the symptoms of pulmonary catarrh, in hooping-cough, did not consider this disease as a simple phlegmasia: he describes it in his work under the name of convulsive catarrh, and openly proclaims its eminently nervous nature. If some physicians have fallen into the error of regarding hooping-cough as an ordinary inflammation only, it is not auscultation which has led them into it; but it is merely by judging the question with a determination to make this morbid affection agree with that exclusive theory, which considers inflammation the sole source of our diseases.

5th. Auscultation practised with care, and above all combined with percussion, will enable us to recognise in tubercles, existing as yet in the latent state in the pulmonary parenchyma, the source of the general symptoms, such as fever, emaciation, anorexia, etc., which otherwise could not be accounted for. Nothing, indeed, is more common than latent phthisis; it is even probable that pulmonary tubercles do not manifest their presence by cough and dyspnœa, until some time after their appearance. But one thing, altogether inexplicable, and of which a doubt might exist if auscultation did not furnish the proof, is, that sometimes they commence by exciting general symptoms, before any local symptom reveals their existence. Hence the precept for every prudent practitioner to auscult all his patients, or at least those who afford the slightest foundation for a suspicion of tubercles.

6th. To auscultation, as much at least as to pathological anatomy, is due the knowledge of this fact, until then doubtful, the possibility of the cure of phthisis pulmonalis, at all events for a longer or shorter time. It is by the aid of this powerful means of investigation, that Laennec has ascertained that scarcely any phthisical patient dies with the first attack of this terrible disease; that the caverns excavated in the pulmonary parenchyma by the softening and evacuation of tubercles, persist in some instances for a great length of time, the patient preserving in other respects every appearance of health, and the autopsy subsequently showing them lined with a false membrane, which in time becomes almost cartilaginous, and forms a kind of wall protecting the parenchyma, which without it would have been exposed. But it must be granted, that this knowledge has not brought such satisfactory results to humanity as were of right expected. The certainty that now exists of the possibility of the cicatrisation of tubercular cavities, is well compensated for by another fact, not less absolute, that there is ordinarily and with almost all patients, a secondary irruption of tubercles, which will soften like the preceding and form new cavities in the parenchyma, will render hæmatosis still more difficult, and thus produce hectic fever and its terrible consequences.

7th. Thus far I have enumerated the advantages which have accrued to the diagnosis of phthisis pulmonalis and catarrh, from the discovery of Laennec: it still remains to be shown what are those which the therapeutics of these two diseases have received. It is evidently a great advantage, in the treatment of pulmonary catarrh, to determine that the bronchial mucous membrane is alone affected. What confidence does it give the practitioner! How much more fearless must he be in employing methods of treatment, to the success of which he knows in advance that no serious organic alteration is opposed! In the case of phthisis perhaps a contrary effect has resulted, and it is difficult for the practitioner not to be discouraged when he has ascertained beyond all doubt that the patient who asks health at his hands, is attacked with pulmonary tubercles. But, in truth, can the reproach be cast upon auscultation? It is not the certainty of the evil which increases it, and it seems to me always better to know the gravity of the danger which the patient runs, than to remain in an uncertainty which is nowise advantageous to him.

Besides, auscultation and percussion combined (for the results, when separated, are as imperfect as they are useful when united), by enabling us to determine precisely the seat of pulmonary caverns, have furnished the idea of therapeutic means of which, without them, we should never have dreamed. It is thus that M. Piorry has tried to treat phthisis by compression of the chest, made on a level with the tubercular cavities, by means of a pyramid formed of compresses, and maintained by a roller. The results which he pretends to have obtained, are: the immediate suppression of the pectoriloquy, which is replaced by the natural respiratory murmur; a greater facility of expectoration, and at the same time a notable diminution of the matter expectorated. (See *Bulletin clinique* for June, 1836, and *Gazette Médicale,* 1836, p. 476). I have not heard that this method was generally attended with success; but it is an experiment which without auscultation would never have been made, and it is well that in such a disease as phthisis pulmonalis we can repeat this aphorism: *Melius est anceps remedium quàm nullum.*

But in auscultation as in all things human, it is difficult for the encomiums not to be tempered by some objections; and I think that as regards phthisis, some just ones can be made to this otherwise admirable discovery. Occurring at a time when pathological anatomy, cultivated with more ardor than ever, was rapidly marching to its avowed end, of *localising* all diseases, and of striking general affections from nosological tables, auscultation, by enabling the progress of pulmonary disorganisation to be followed from to day, has still more favored this vicious tendency of the medical spirit of our time. Since its introduction, in the view of many physicians, phthisis has been a mere local disease, limited to the lung, and affecting the rest of the organism by sympathy only. How many powerful objections, however, are to be made to this theory! Why is the hectic fever not at all in proportion to the extent of the lesions presented by the lung? Why do we see consumption and marasmus in their last stage, accompanying small pulmonary cavities, in persons who yet have a very large portion of healthy parenchyma, whilst others, whose lungs are three fourths destroyed, still survive for a very considerable time? Why this constant coincidence of lesions of the larynx, of the stomach, and of the intestines, with destruction of the lungs by tubercles? Is it sympathy of the lung with those organs that causes it? But then, why does not acute pneumonia provoke these sympathies? Lastly, how can we explain the simultaneous developement of tubercles in almost all the organs; and this law, which, without being so constant as M. Louis thought when he promulgated it, is nevertheless often confirmed by experience: *that tubercles are never found in any organ, without they exist at the same time in the lungs?*

Phthisis pulmonalis is not then a local disease, but it affects at the same time the entire organism. It results from a morbid action which goes on in the whole body at the same time; a profound alteration of nutrition appears to be its cause, and the ancients were not wrong in describing a *tubercular constitution.* Lastly, men of superior minds have not failed to protest against this unhappy tendency, given to the study of phthisis by auscultation; and Dr. James Clark published at London, in 1834, a *Treatise on Consumption or Tubercular Phthisis,* whose object was to bring back practitioners to the study of the *tubercular constitution and cachexia* respecting which he puts forth some eminently judicious opinions, but which it does not enter into my plan to develope here.

### CHAPTER III.—OF PNEUMO-THORAX.

The present chapter will serve in some degree as an appendix to the two preceding, for the disease we are now considering is in fact almost always the result of a chronic pleurisy, or of phthisis pulmonalis. Under the name of pneumo-thorax, is designated an accumulation of gas or of elastic fluids in the thoracic cavities. Do the cavities of the pleura contain any in the natural state? the general opinion is that they do not. However, Laennec thought with M. Ribes that the pleuræ, in the healthy state, might contain a small quantity, arising from the vaporisation of the liquid which constantly lubricates their internal surface. Be this opinion as it may, it is certain that this gas, if it exists, is always inappreciable in autopsies. But it sometimes happens that the pleura is so filled that the accumulated gas forces the lung back towards its root, and distends the parietes of the thorax in a very perceptible manner.

This disease, although far from being rare, had not attracted the attention of any author, until M. Itard, now dead, physician to the deaf and dumb, chose it for his inaugural dissertation. But if the honor of its first description does not belong to Laennec, that of having first fixed the diagnosis in a certain manner, which we shall soon prove to be extremely difficult, if not impossible without auscultation, will not be denied him.

A gas may be found in the pleura, even in great quantity, without there being the slightest solution of continuity, the least alteration of this membrane, or any effusion whatever into its cavity. But this is not the most common case. Nearly always there is at the same time an effusion of liquid into the pleura; whether this liquid be pus, as it most frequently is, or serum, as in hydro-thorax, or blood coming from a penetrating wound of the chest, or lastly, tubercular matter from a vomica opening into the pleura; in all these cases, save the last, we are forced to admit, that the gas contained in the pleura is the product of the putrid decomposition of the effused liquids, an origin which the fetidity of its odor often induces us to suspect. When the effusion consists of tubercular matter from a cavity, if the latter does not communicate with the bronchiæ, we are yet forced to attribute the production of the gas to the decomposition of the effused matter. But if, which is more common, we find in this cavity the orifices of several bronchial ramifications, there is no longer a doubt that the gas is nothing more than atmospheric air which has passed into the pleura.

We have already seen that this affection had been almost forgotten by the majority of authors anterior to Laennec; regarding it only as a rare phenomenon, of little importance, no endeavors were made to determine its peculiar symptoms. Had this been attempted, however, success would have been impossible. How, indeed, could general symptoms, so indefinite as those drawn from the state of the pulse, from the inspection of the tongue and of the excretions, have served for the recognition and discrimination of the numerous diseases with which the chest may be attacked? In this respect, therefore, the method proposed by Avenbrugger, and so ably developed and applied by Corvisart, made an immense step in pathology, and opened the way for the improvement which auscultation was to effect in the same science.

Percussion alone, however, could not in all cases distinguish hydro-thorax in a precise and indubitable manner. Where this affection exists in a high degree, the progressive augmentation of the clearness of the sound in the side primarily affected, may indeed cause it to be suspected; but a source of grievous error has to be guarded against. The greater dulness on the healthy side may make that be regarded as unsound which is in a normal state, and as healthy, that precisely which is the seat of disease. Still, this mistake could not be committed, save by one who had not followed the disease in its whole course, and besides, the pain existing in the side affected with pneumo-thorax would in most cases prevent it.

M. Piorry, in the article relative to this disease, in the *Dictionnaire des Sciences Médicales,* has proposed, as a very excellent means of detecting the complication of pneumo-thorax with an effusion in the pleura, to percuss the patient in different positions. The liquid, being the heavier, makes its way to the lowest part, and the sound is successively clear and flat in the same portion of the chest. But the application of this method is difficult in the majority of cases; and to obtain the most conclusive results possible, it would be necessary to percuss the patient first in the vertical position and then in the reverse situation, in order that the summit of the chest should for a moment become the base, which is morally impossible. Lastly, if there existed adhesions, percussion might lead into error. This is true, but it should be added, that these adhesions would become sources of error in all other methods, even in auscultation.

Ought we to rely more on abdominal pressure, proposed by Bichat and still lauded by M. Piorry, to aid the diagnosis of thoracic diseases? It is certain that, all things equal, abdominal pressure is incomparably more painful for patients suffering under an effusion of air or of water in the chest, than for those who have an inflamed lung only. But what vague information does such a means furnish! What uncertainty would a diagnosis have, founded on such a basis! Besides, abdominal pressure will be more or less easily practised, not only according to the greater or less vacuity of the chest, but also according to the greater or less sensibility of the patient. And finally, had this means all the efficacy that its author attributes to it, it would never serve to indicate the nature of the fluid which fills the pleura, and could not make known whether it was a gas or a liquid which prevented the diaphragm from being carried up, by abdominal pressure, as far as it would go in the natural state.

Auscultation alone, then, can furnish information sufficiently certain to enable us to recognise pneumo-thorax in the plurality of cases, if not in all: this will be easy for me to prove.

We must distinguish two species of this affection: 1. pure pneumo-thorax, that is, the simple accumulation of gas in one of the cavities of the chest, without effusion in the pleura; 2. pneumo-thorax complicated with effusion.

Pneumo-thorax without effusion is extremely rare, but yet a few cases have been reported: (see Obs. xxxvi of Laennec, vol. ii, p. 249). It will be known by the affected side yielding a much clearer sound on percussion than the healthy one, by the complete absence of the respiratory murmur in the diseased side, the other continuing to present it as usual. Thus absence of the respiratory murmur coinciding with increased sonorousness of the chest, is the pathognomonic sign of simple pneumo-thorax. Pulmonary emphysema, it is true, presents analogous symptoms, and particularly the same coincidence; but in emphysema, the respiratory murmur is never so completely absent as in pneumo-thorax: and if it may sometimes appear so, it will then be wanting in the whole extent of the diseased side of the chest without exception; whilst in pneumo-thorax, however considerable may be the quantity of gas accumulated in the pleura, the respiratory murmur may always be heard along the vertebral column, a point where the compressed lung is found, precisely as we have seen it in hydro-thorax. Besides, pulmonary emphysema is developed slowly, and may exist in a high degree, without the patient ceasing to follow his occupation; whilst pneumo-thorax comes on suddenly, attains at once a considerable developement, and occurs only in bed-ridden patients already weakened by long suffering.

Pneumo-thorax with effusion, is, as we have said, the most frequent of all. When it exists, whether complicated or not with pulmonary fistula, and with a communication between the cavity of the pleura and the bronchiæ, it is the only case of effusion to which can be applied the exploratory method advised by Hippocrates for detecting empyema: I mean the succussion of the chest. It is certain that when pus is contained in the pleura without occupying its whole capacity, the rest being filled by an accumulation of gas or of atmospheric air, succussion of the chest will most often produce a sound easily recognised by the stethoscope, and which is sometimes heard by those standing near. We may read in Laennec the learned dissertation which he has given on this method of the Asclepiades, which so long remained in utter neglect, because it was not understood by the various commentators of Hippocrates. Almost all the authors who have studied this passage, have regarded the method as impracticable, and above all as affording no result. This opinion, true as it respects ordinary effusions, is evidently false so soon as it refers to hydro-pneumothorax: and it is an established fact, that to hear the noise of fluctuation in the chest, is a proof that this cavity contains gas, besides the effused matter; for the liquid can not fluctuate except in a space filled with air.

But this sign, however important it may be, is fortunately not the only one by which the lesion we are studying may be recognised. Laennec has indicated two others, which, when they exist, place it beyond a doubt, that air and pus are simultaneously contained in one of the cavities of the pleura. These are, the *amphoric respiration* and the *metallic tinkling*. Laennec thought that metallic tinkling could take place only in cases of fistulous communication of the bronchiæ with the cavity of the effusion; but it appears, from some rare, though well observed cases, that this condition is not indispensable: it is so, however, for the amphoric respiration, whose presence denotes necessarily the communication of the bronchiæ with the pleura.

What is the cause of the metallic tinkling? Here we find a perfect confusion among authors, all of whom attribute it to a different source. Laennec thought it was owing to the fall of a drop of liquid from the upper wall of the chest back on the effusion below, when the patient rises suddenly in his bed. His opinion was founded on the circumstance that a real metallic tinkling is heard whenever a drop of water falls from a small height into a half-filled decanter. To him the similarity of the results proved the analogy of the causes.

M. Beau, in an article inserted in the *Archives générales de médicine* (March 1834), has proposed another theory for the metallic tinkling. This physician thinks that this really characteristic noise is always produced by the rupture of an air bubble in the midst of a thoracic, pleural or cavernous effusion. According to him, it can be produced by this cause only; but as certain patients sometimes present this noise several days in succession and without interruption, it may be asked, what would become of the enormous quantity of gas which would always be accumulating in the pleura, if the proposition were true.

The opinion of Laennec, founded on fact, could evidently not be applied to all cases of metallic tinkling. That of M. Beau, true also in some circumstances, can no more pretend to the honor of explaining all the cases. Therefore many authors have admitted both, as accounting for the various circumstances which favor the production of this noise. But some have indicated other circumstances as indispensable to the occurrence of this phenomenon. Thus, Mr. Guthrie affirms that the presence of a certain quantity of compressed air is a *sine quâ non* for the production of the metallic sounds. Dr. Thomas Davies thinks that this air must be agitated at the surface of the effused liquid; and Dr. Houghton regards the sounds as a mere echo, which is itself produced by the fall of a liquid in the cavity, and the entrance of air through a fistulous opening.

Other authors have rejected all these theories, to substitute others which do not appear to me even so admissible. Among these I will cite only Dr. Bigelow, who, in his remarks on pneumo-thorax in the 4th number of *The American Journal of Medical Sciences*, for 1838, after having stated all the reasons which prevented him from admitting the theories of Laennec and others whom I have cited, lays down as principles, resulting from the experiments performed by himself: 1st. that "the immediate or exciting cause of metallic tinkling, is a sudden and violent commotion of the liquid in a vibrating cavity . . . . . , the same phenomenon taking place when a part of the liquid is thrown up by the cough, and then falls back on the rest:" (is this not a return to the falling drop of Laennec?); 2dly. "the amphoric resonance is produced by the reverberation of the air in a vibrating cavity, and without the sonorous impulsion of the liquid, etc."

However it may be with these various explanations, let us note as a fact of great importance that the value of the metallic tinkling has not changed, and that it is always the pathognomonic sign of an effusion of air and liquid into the same cavity, this cavity almost always communicating with the bronchiæ. I ought, however, to state, that the metallic tinkling and the amphoric respiration may also present themselves in the case of a very large, but nearly empty pulmonary cavity. But, even in this case, "it is difficult to be mistaken," says Laennec; "a remnant of pectoriloquy, the small extent of the space in which the metallic tinkling, the amphoric resonance, and the tympanitic sound given by percussion are heard, and the absence of fluctuation, characterise a vast pulmonary cavity; the cough, on the other hand, sometimes produces a gurgling or a slight fluctuation which it never does in pneumo-thorax." (Laennec, op. cit., vol. ii, p, 357).

After what I have just said of the new signs furnished by auscultation in the diagnosis of pneumo-thorax, it will not be difficult for me to tell how much this discovery has advanced science on this point: it has enabled us to detect during life an affection which formerly was never perceived before death, and even when recognised on the dead body, was considered as a cadaveric phenomenon only, an occurrence of the dying moments. Laennec says in his work, that whilst he was following the lectures of Corvisart he several times saw him make examinations of subjects affected with pneumo-thorax, in none of whom had this affection been suspected. "No one will deny," he adds, "to this celebrated professor either the talent for observation or the ability to take advantage of percussion; and consequently the best proof which can be given of the insufficiency of this method of recognising pneumo-thorax, is that the latter was not detected in these cases." (Laennec, op. cit., vol. ii, p. 259). Certainly an affection unperceived by Corvisart is of difficult diagnosis; and it has not been a small service rendered to science, to make this diagnosis easy to all.

If it should be asked what advantage has resulted from it to the therapeutics of this disease, I answer, that it is already a very great one to have fixed the nature of the lesion with which the patient who claims our aid is affected. Now, precision of diagnosis is necessarily an approximation to a more efficacious mode of treatment; and though we have not yet attained the desired end, it is nevertheless a great good to know the extent of the evil which we wish to avert. But, if we reflect that pneumo-thorax is most frequently a mode of termination of a disease generally fatal, either chronic pleurisy or phthisis pulmonalis; if we consider that it is most generally a phenomenon of the last stages of the disease, we will see that it was quite natural that so grave a case should present few therapeutic indications, and we shall be less disposed to render auscultation responsible for the little progress which science has made with respect to its treatment.

(TO BE CONTINUED.)

